# Errata

**Published:** 1809-03

**Authors:** 


					In the paging oj the iluurfing litles, Jitter 11, jor 32o?340, read S2p
?240.

				

